# Cancer progression by the okadaic acid class of tumor promoters and endogenous protein inhibitors of PP2A, SET and CIP2A

**DOI:** 10.1007/s00432-023-04800-4

**Published:** 2023-04-25

**Authors:** Hirota Fujiki, Eisaburo Sueoka, Tatsuro Watanabe, Atsumasa Komori, Masami Suganuma

**Affiliations:** 1 Department of Clinical Laboratory Medicine, Faculty of Medicine, Saga University, Nabeshima, Saga 849-8501 Japan; 2 Department of Drug Discovery and Biomedical Sciences, Faculty of Medicine, Saga University, Nabeshima, Saga 849-8501 Japan; 3 Clinical Research Center, National Hospital Organization Nagasaki Medical Center and Department of Hepatology, Nagasaki University Graduate School of Biomedical Sciences, Omura, Nagasaki 856-8562 Japan; 4 Department of Strategic Research, Graduate School of Science and Engineering, Saitama University, Saitama, 338-8570 Japan

**Keywords:** Cancer progression, CIP2A, Okadaic acid, PP2A, SET, TNF-α

## Abstract

**Purpose:**

Okadaic acid class of tumor promoters are transformed into endogenous protein inhibitors of PP2A, SET, and CIP2A in human cancers. This indicates that inhibition of PP2A activity is a common mechanism of cancer progression in humans. It is important to study the roles of SET and CIP2A vis-à-vis their clinical significance on the basis of new information gathered from a search of PubMed.

**Results and discussion:**

The first part of this review introduces the carcinogenic roles of TNF-α and IL-1, which are induced by the okadaic acid class of compounds. The second part describes unique features of SET and CIP2A in cancer progression for several types of human cancer: (1) SET-expressing circulating tumor cells (SET-CTCs) in breast cancer, (2) knockdown of CIP2A and increased PP2A activity in chronic myeloid leukemia, (3) CIP2A and epidermal growth factor receptor (EGFR) activity in erlotinib sensitive- and resistant-non-small cell lung cancer, (4) SET antagonist EMQA plus radiation therapy against hepatocellular carcinoma, (5) PP2A inactivation as a common event in colorectal cancer, (6) prostate cancer susceptibility variants, homeobox transcription factor (*HOXB13* T) and *CIP2A* T, and (7) SET inhibitor OP449 for pre-clinical investigation of pancreatic cancer. In the Discussion, the binding complex of SET is briefly introduced, and overexpression of SET and CIP2A proteins is discussed in relation to age-associated chronic inflammation (inflammaging).

**Conclusion:**

This review establishes the concept that inhibition of PP2A activity is a common mechanism of human cancer progression and activation of PP2A activity leads to effective anticancer therapy.

## Introduction

**Fig. 1:**
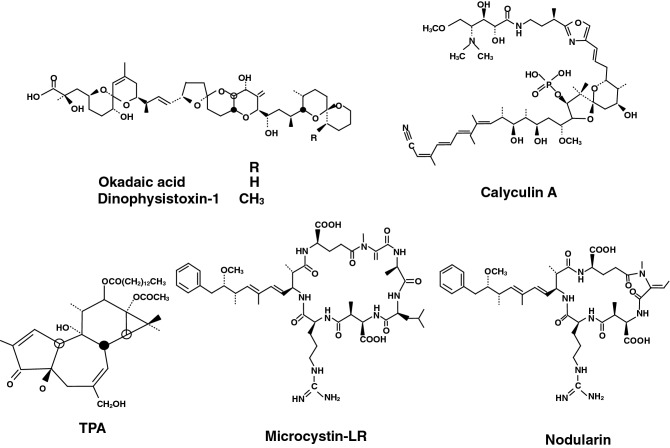
Structures of TPA, okadaic acid, dinophysistoxin-1, calyculin A, microcystin-LR, and nodularin

Okadaic acid is a polyether compound of a C_38_ fatty acid, which is isolated from the black sponge, *Halichondria okadai* (Tachibana et al. 1981). Okadaic acid acts as a tumor promoter in mouse skin initiated with 7,12-dimethylbenz(a)anthracene (DMBA) and is as potent as 12-*O*-tetradecanoylphorbol-13-acetate (TPA) (Table 1, Fig. 1) (Suganuma et al. 1988; Hecker et al. 1967). Because okadaic acid is a chemical inhibitor of serine /threonine protein phosphatase 1 and 2A (PP1 and PP2A) (Bialojan and Takai 1988), the mechanism of okadaic acid is different from activation of protein kinase C by TPA (Castagna et al. 1982). Utilizing an assay based upon inhibition of [^3^H]okadaic acid binding to the particulate fraction of mouse skin, we identified other compounds of the okadaic acid class as follows (Fujiki and Suganuma 1993): dinophysistoxin-1 (35-methylokadaic acid), isolated from the mussel *Mytidus edulis* (Murata et al. 1982); calyculin A, isolated from a marine sponge *Discodermia calyx* (Kato et al. 1986); and microcystin-LR and nodularin, isolated from toxic blue-green algae *Cyanobacteria* (Harada et al. 1988) (Fig. 1). Moreover, okadaic acid is a more effective inhibitor of PP2A (50% inhibitory concentration, IC_50_ = 0.07 nM) than PP1 (IC_50_ = 3.4 nM), and other compounds of the okadaic acid class are equally effective against the purified catalytic subunits of PP1 and PP2A (IC_50s_ = 0.1 to 0.7 nM) (Suganuma et al. 1992a). Later, the compounds were additionally found to inhibit PP4 (IC_50_ = 0.1 to 0.4 nM) and PP5 (IC_50_ = 1.0 to 10 nM), which also belong to the class of serine/threonine protein phosphatases (Honkanen and Golden 2002).

**Table 1:** Tumor-promoting activity of TPA and the okadaic acid class of compounds

Tumor promoter	Initiator	Target organ	Tumor	References
TPA	DMBA	Mouse skin	Papilloma	Hecker et al. (1967)
Okadaic acid	DMBA	Mouse skin	Papilloma	Suganuma et al. (1988)
Dinophysistoxin-1	DMBA	Mouse skin	Papilloma	Fujiki et al. (1988)
Calyculin A	DMBA	Mouse skin	Papilloma	Suganuma et al. (1990)
Okadaic acid	MNNG	Rat glandular stomach	Neoplastic changes with adenocarcinoma	Suganuma et al. (1992b)
Microcystin-LR	DEN	Rat liver	GST-P positive foci	Nishiwaki-Matsushima et al. (1992)
Nodularin	DEN	Rat liver	GST-P positive foci	Ohta et al. (1994)

*TPA* 12-*O*-tetradecanoylphorbol-13-acetate, *DMBA* 7,12-dimethylbenz(a)anthracene, *MNNG* *N*-methyl-*N’*-nitro-*N*-nitrosoguanidine, *DEN* diethylnitrosamine, *GST-P* glutathione *S*-transferase placental form

Tumor promoter induces clonal growth of initiated cells. As Table 1 shows, okadaic acid, dinophysistoxin-1, and calyculin A are potent tumor promoters in mouse skin. In addition, okadaic acid in drinking water induced tumor-promoting activity in rat glandular stomach initiated with *N*-methyl-*N’*-nitro-*N*-nitrosoguanidine (MNNG) (Table 1), and repeated intraperitoneal (i.p.) injections of microcystin-LR or nodularin induced potent tumor-promoting activity in rat liver initiated with diethylnitrosamine (DEN) (Table 1). Thus, inhibition of PP1 and PP2A activities by the okadaic acid class of compounds induces tumor-promoting activity in mouse skin, rat glandular stomach, and rat liver, each treated with a different initiator (Table 1). These results demonstrate that inhibition of PP1 and PP2A activities is a general mechanism of tumor promotion in various organs of rodents (Fujiki and Suganuma 1993).

Fearon and Vogelstein presented a multi-stage model of carcinogenesis in human colorectal tumorigenesis; their model consists of putative initiation (mutation), tumor promotion, and progression, the latter of which produces conversion from adenoma to carcinoma with numerous genetic changes (Fearon and Vogelstein 1990). In our previous article in *Adv Cancer Res* (Fujiki and Suganuma 1993), we considered how the okadaic acid pathway is related to human cancer, and pointed out two key points: effects of the okadaic acid class of compounds can be mimicked by those of cytokines such as tumor necrosis factor-α (TNF-α) and interleukin-1 (IL-1) (Guy et al. 1992), and the inhibitory effect of small-t antigen on dephosphorylation by PP2A could explain its role in transformation (Scheidtmann et al. 1991). Thus, the inhibition of protein phosphatase activity commonly induces not only cell autonomy but also a non-autonomous effect, the latter of which is related to endogenous inflammation produced by proinflammatory cytokines; both of these induce tumor development (Fujiki 1992).

In 1992, the *set-can* fusion gene was first found in acute undifferentiated leukemia, and the SET protein is estimated to have mass 32 kDa with 277 amino acids (von Lindern et al. 1992). I_2_^PP2A^ isolated from extract of bovine kidney was homologous to SET, an inhibitor of PP2A (IC_50_ = 2.0 nM) (Li et al. 1995). CIP2A, an inhibitor of PP2A in cancer, was first identified as a 90 kDa protein in human hepatocellular carcinoma, (Soo Hoo et al. 2002). Before going into the mechanisms of SET and CIP2A, it is important to note that PP2A accounts for dephosphorylation of ~ 55—70% of all serine/threonine-protein phosphatase and dysregulation of protein kinase signaling is hallmark of many diseases in which an increase in oxidative stress is also noted (Elgenaidi and Spiers 2019). Numerous scientists have reported an overexpression of SET and CIP2A proteins in various types of human cancers, and the knockdown of SET or CIP2A proteins in cancer cells after transfection with SET- or CIP2A-targeted siRNA and shRNA resulted in a reduction of SET or CIP2A levels and inhibited tumor growth. Moreover, SET antagonists, such as OP449 and FTY720 inhibited proliferation of cancer cells. This suggests that overexpression of SET and CIP2A induces strong inhibition of PP2A activity in human cancer cells, as okadaic acid does (Fujiki et al. 2018a). This review describes first the mechanisms of cancer progression by the okadaic acid class of compounds and second those by the SET and CIP2A proteins.

## Carcinogenic role of proinflammatory cytokines induced by the okadaic acid class of compounds

After topical application of okadaic acid or TPA to the skin on the backs of mice, *TNF-α* gene expression was commonly induced dose-dependently 4 h later (Fujiki et al. 2000). In two-stage carcinogenesis experiments with mouse skin, TNF-α^−/−^ mice treated with DMBA plus okadaic acid showed no tumors for up to 19 weeks, whereas in similarly treated TNF-α^+/+^ mice the percentage of tumor-bearing mice was 100%. Moreover, residual tumor-promoting activity in the skin of TNF-α^−/−^ mice was associated with induction of *IL-1α* and *IL-1β* gene expressions (Suganuma et al. 1999). Furthermore, single administration of a liver tumor promoter, microcystin-LR or nodularin, induced expression of *TNF-α* and early-response genes in primary cultured rat hepatocytes (Sueoka et al. 1997), whereas administration of TPA, a non-liver tumor promoter, induced neither *TNF-α gene* expression in primary cultured rat hepatocytes nor *c-jun* gene expression in rat liver (Sueoka et al. 1996). Thus, proinflammatory cytokines, such as TNF-α and IL-1, are induced in target organs of rodents by treatment with chemical tumor promoters. Since human TNF-α is inflammatory and has possible tumor-promoting activity, we studied the initiating activity of human TNF-α as well. Treatment of BALB/3T3 cells with human TNF-α at a concentration of 10 ng/ml (0.6 nM) alone induced an average of 0.33 foci/dish, whereas 3-methylcholanthrene (MCA) at a concentration of 0.1 μg/ml (5.9 nM) alone induced 0.08 foci/dish. These experiments demonstrate that human TNF-α is a carcinogen because it possesses both initiating and tumor promoting activities. The carcinogenic potential of human TNF-α was also studied by using v-Ha-*ras* transfected BALB/3T3 (Bhas 42) cells. Human TNF-α strongly induced growth of Bhas 42 cells, whereas it did not induce growth of non-transfected BALB/3T3 cells due to the absence of the v-Ha-*ras* gene. Furthermore, clones from the human TNF-α-transformed foci of Bhas 42 cells induced tumorigenicity with *IL-6*, *TGF-β*, and *IL-1α* gene expressions at sites of injection of mice, suggesting that human TNF-α and other proinflammatory cytokines serve as essential carcinogenic factors in endogenous inflammation (Komori et al. 1993). Recently, an orally active, small-molecule TNF inhibitor (TNF-inhibitory molecule 1, TIM1) was found to be effective for treatment of rheumatoid arthritis and other TNF-dependent systemic disorders of inflammation (Javaid et al. 2022), and TIM1 seems to be useful in cancer treatment. Next, we discuss cancer progression induced in various organs by SET and CIP2A.

## Mechanisms of cancer progression related to SET and CIP2A in several types of human cancer

### Breast cancer

**Fig. 2:**
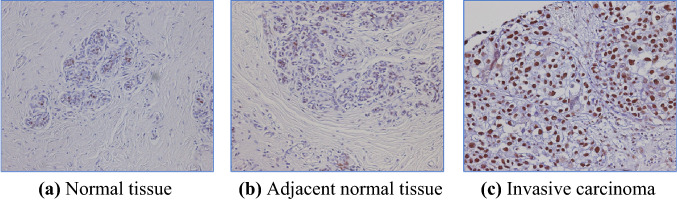
Immunohistochemical analysis of a breast cancer tissue array. **a** normal tissue, **b** adjacent normal tissue, and **c** invasive carcinoma. SET protein was overexpressed in invasive carcinoma tissues compared to normal and adjacent normal tissues (Tozuka et al. 2021)

**Fig. 3:**
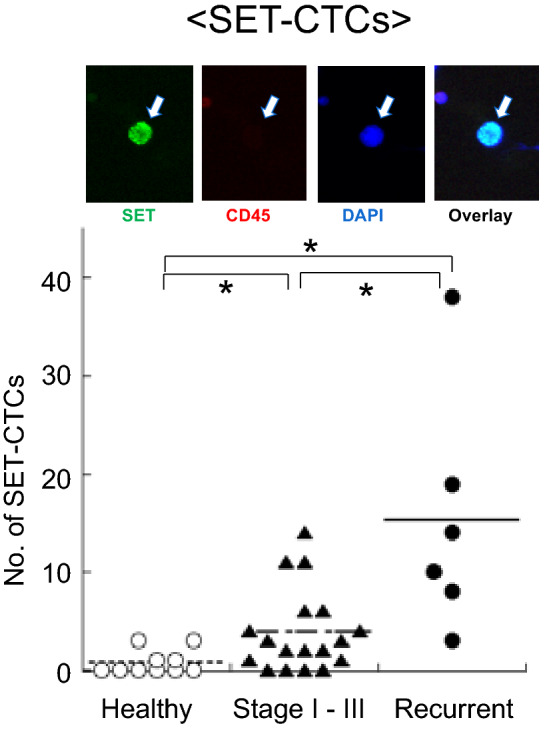
Immunocytochemical analysis of SET-CTCs in blood of breast cancer patients. Staining of the nucleus with the anti-SET antibody (green), along with 4',6-diamidino-2-phenylindole (DAPI) staining, but not with anti-CD45 antibody (absence of red). The number of SET-CTCs differed significantly between healthy controls and patients with stage I–III disease, between healthy controls and patients with recurrent disease, and between patients with stage I–III disease and patients with recurrent disease (Tozuka et al. 2021) *P < 0.05

SET is a nuclear protein, hence strong overexpression of SET was detected in the nuclei of invasive breast carcinoma tissue. Immunohistochemical staining of breast tissue arrays revealed that SET protein was significantly overexpressed in invasive carcinoma tissues when compared to normal and adjacent normal tissues (Fig. 2). Recently the presence of SET-expressing circulating tumor cells (SET-CTCs) in 24 breast cancer patients were analyzed using an anti-SET antibody, along with 4',6- diamidino-2-phenylindole (DAPI) staining. SET-CTCs were detected in 6/6 (100%) patients with recurrent breast cancer with a median value of 12 (12 cells/3 ml blood) and in 13/18 (72.2%) patients with stage I-III breast cancer with a median value of 2.5, while the median value of healthy controls was 0 (Fig. 3). The relationship between the number of SET-CTCs and lymph node metastasis among patients with stage I-III disease revealed that a large number of SET-CTCs is correlated with lymph node metastasis in breast cancer patients (Tozuka et al. 2021). Since overexpression of SET and CIP2A induces cancer progression, and knockdown of SET and CIP2A inhibits tumor growth, the effects of anticancer drugs might be different in between cancer cells with SET and CIP2A and those with their knockdown. Tamoxifen is the first cancer preventive agent, with 50% prevention of primary breast cancer development in senior women and a high-risk group (Fisher et al. 1998). Now tamoxifen is used as an adjuvant to reduce recurrence of estrogen receptor-positive breast cancer. The overexpression of SET suppressed tamoxifen-induced anti-cancer effects and upregulated estrogen receptor element (ERE)-dependent ER signaling transactivation, indicating that SET may be associated with the failure of tamoxifen treatment in ER-positive breast cancer MCF-7 cells (Huang et al. 2018b). Doxorubicin is the most effective anthracycline antibiotic, but nearly 50% of breast cancer patients had treatment failure due to growing resistance to doxorubicin. The PP2A activity in MCF-7/ADR cells after doxorubicin treatment and silencing *CIP2A* was studied. PP2A activity was induced by doxorubicin alone, while *CIP2A* knockdown further enhanced PP2A activity to a greater level, even in the presence of doxorubicin. In addition, downregulation of *CIP2A* induced the autophagy markers, LC3B and Beclin1, at protein level in MCF-7/ADR cells. Thus, the results support the potential benefits of CIP2A inhibition for breast cancer treatment via activity of PP2A (Zhu and Wei 2021). SET, CIP2A, and pS62-MYC proteins are commonly overexpressed in human breast cancers. Treatment of MDA-MB-231 cells with one of SET antagonists, OP449, and SET or CIP2A knockdown caused a decrease in pS62-MYC protein and MYC binding to the promoters of its target genes, nucleolin, E2F2, and 5s rRNA. These results suggest that inhibiting either SET or CIP2A could be a successful strategy to target MYC post-translationally and inhibit tumor growth in breast cancer (Janghorban et al. 2014). Another, more direct piece of evidence was recently reported: novel Myc-binding compound MYCMI-7 showed efficacy toward MDA-MB-231 cells, which are derived from triple-negative breast cancer (Castell et al. 2022).

### Chronic leukemia

CIP2A levels are significantly higher in chronic myeloid leukemia (CML) patients who later progress to blast crisis than in patients who do not. High CIP2A levels in primary CML cells were correlated with high levels of pS62-Myc, and serine/threonine-protein kinase pim-1 (PIM1) can phosphorylate and stabilizes c-Myc. CIP2A expression regulated by BCR-ABL1 activity has a role in suppression of PP2A activity and in downstream activation of PIM1 and c-Myc. Activation of PP2A, either by addition of a PP2A activator or inhibition of CIP2A by imatinib, also decreased PIM1 levels. The role of PP2A in regulating PIM1 protein levels suggests that PP2A activity is suppressed in the CD34^ +^ cells of patients at high risk of developing blast crisis. Knockdown of CIP2A resulted in increased PP2A activity, decreased c-Myc levels, and a decrease in BCR-ABL1 tyrosine kinase activity. The BCR-ABL1 tyrosine kinase in CML is responsible for growth and survival of the malignant cells through activation of signaling pathways such as mitogen-activated protein kinase cascade and the phosphatidylinositol-3 kinase (PI3K) pathway (Lucas et al. 2011). Using the UK SPIRIT 2 (STI1571 Prospective International RandomIsed Trial 2) clinical trial, CIP2A was validated as a diagnostic biomarker to identify patients at risk of disease progression and treatment failure (Clark et al. 2021).

### Lung cancer

Non-small cell lung cancer (NSCLC) harboring activating mutations in the epidermal growth factor receptor (EGFR) tyrosine kinase domain are typically treated with the EGFR tyrosine kinase inhibitor, erlotinib. Recently, the effects of SET and CIP2A proteins on the mutations and drug resistance of cancer cells have provided new insights into cancer therapy. For example, the effects of CIP2A protein level on EGFR activity was studied in cells of the NSCLC cell line HCC4006 and in HCC4006^r^Erlo^0.5^ cells. In HCC4006 cells, EGF stimulation did not affect CIP2A levels, but EGFR inhibition by erlotinib markedly reduced the amount of cellular CIP2A, which resulted in PP2A activation followed by complete loss of Akt phosphorylation. CIP2A has been shown to activate Akt signaling via inhibition of Akt dephosphorylation in different types of cancer, including lung cancer. In HCC4006^r^Erlo^0.5^ cells with resistance to erlotinib, neither EGF nor erlotinib affected CIP2A protein levels or PP2A activity. Similarly, EGFR inhibition resulted in a slight decrease of p53 and a robust increase in the cell cycle regulator p27 in HCC4006 cells, whereas these proteins were completely unaffected in HCC4006^r^Erlo^0.5^ cells. The proteasome inhibitor bortezomib reduced CIP2A protein level with accompanying reduction of the PP2A inactivity marker pY307 and a reduction of pAkt in both HCC4006 cells and HCC4006^r^Erlo^0.5^ cells. Similarly, bortezomib markedly induced the cell cycle inhibitors p21 and p27 and cell cycle arrest in G_2_/M. In addition, the magnitude of effect by bortezomib increased in HCC4006^r^Erlo^0.5^ cells, compared to the erlotinib-sensitive HCC4006 cells (Saafan et al. 2021).

### Hepatocellular carcinoma

The proteasome inhibitor bortezomib down-regulated CIP2A in a dose- and time-dependent manner in all sensitive hepatocellular carcinoma (HCC) cell lines, whereas no alterations in CIP2A were found in resistant PLC5 cells. For example, bortezomib down-regulated CIP2A and increased PP2A activity in Huh-7 tumors, but not in resistant PLC5 tumors (Chen et al. 2010). Co-expression of SET and Akt predicted shorter post-operative recurrence-free survival in patients with HCC, and the combination of a novel SET antagonist, *N*^4^-(3-ethynylphenyl)-6,7-dimethoxy-*N*^2^-(4-phenoxyphenyl) quinazoline-2,4-diamine (EMQA developed by Hung et al. 2016), and sorafenib acted synergistically in terms of enhancing survival from HCC (Hung et al. 2016). The mechanism of EMQA action in HCC cells was revealed that EMQA interfered with the interaction of SET and PP2Ac and increased the PP2A activity in the cells. The results are supported by the evidence that downregulation of pAkt and the proapoptotic effects induced by EMQA treatment were diminished by knockdown of PP2Ac. Investigation of the impact of SET on radiation therapy (RT)-mediated anticancer effects, by using colony and hepatosphere formation assays, revealed that RT-induced proliferative inhibition was more prominent in PLC5 cells with SET-knockdown, whereas overexpression of SET had a minimal effect on the radiation-induced DNA damage and repair process, suggesting that expression of SET determines the radiosensitivity of HCC cells. Moreover, EMQA promoted RT-induced apoptosis in various HCC cell lines. Mice bearing PLC5 xenografted tumors were treated with RT plus EMQA, which led to the most significant inhibition of the average tumor growth without affecting tolerability, and to downregulation of pAkt and PP2A reactivation in the xenografted tumors. The effects of RT plus EMQA against HCC provides a clinical benefit for treatment of HCC (Huang et al. 2018a).

### Colorectal cancer

Colorectal cancer arises through an adenoma-dysplasia-carcinoma sequence, in which *APC* and *KRAS* gene mutations occur early and *Tp53* mutation occurs at a late stage. Overexpression of SET in specimens of colorectal cancer obtained from patients indicated that PP2A inactivation is a common event and deregulation of SET would be a key contributing mechanism to PP2A inactivation. A SET antagonist, FTY720, induced PP2A activity in two human colorectal cancer cell lines, RKO and LoVo, and was inhibited by okadaic acid. FTY720 treatment decreased phosphorylation of the PP2A targets Akt and ERK1/2 without affecting their expression levels. Moreover, okadaic acid treatment rescued Akt and ERK1/2 phosphorylation in FTY720-treated RKO cells (Cristóbal et al. 2014). Since SET and CIP2A are commonly overexpressed in human cancers, we think that cancer preventive agents can act by reducing the overexpression of SET and CIP2A. Cancer preventive activities of green tea and its main constituent, (-)-epigallocatechin gallate (EGCG), have been studied extensively by scientists all over the world (Fujiki et al. 2018b). A double-blind randomized clinical phase II prevention trial showed that drinking 10 Japanese-size cups of green tea (120 ml/cup), supplemented with green tea extract, reduced recurrence of colorectal adenoma by 51.6% (Shimizu et al. 2008). A similar clinical trial showed that drinking green tea extract prevented 44.2% of colorectal adenoma recurrence in Korean patients at Seoul National University (Shin et al. 2018). Although interaction of green tea catechin with SET or CIP2A remains to be investigated, numerous scientists reported that human cancer stem cells are a target for cancer prevention using EGCG (Fujiki et al. 2017).

### Prostate cancer

Treatment with the PP2A activators forskolin and FTY720 reduced prostasphere formation capability of two prostate cancer cell lines, PC-3 and LNCaP; however, pretreatment with the PP2A inhibitor, okadaic acid, partially quenched the forskolin- and FTY720-induced antitumor effects. Immunohistochemical staining revealed that CIP2A, SET, and pPP2A were found in a high-risk subgroup of prostate cancer patients and were associated with metastatic potential. These results show that PP2A inhibition status is a critical alteration facilitating progression of prostate cancer (Cristóbal et al. 2015). A homeobox transcription factor (*HOXB13*) is important in prostate development. Dual carriers of *HOXB13* rs138213197 T *and CIP2A* rs2278911 T, both of which are prostate cancer susceptibility variants, show the risk of prostate cancer with threefold higher odds than the *HOXB13* T allele alone. This suggests that the possible interaction of *HOXB13* and *CIP2A* is related to prostate cancer susceptibility (Sipeky et al. 2018). Prostate cancer development in patients with high-grade prostate intraepithelial neoplasia (PIN) was prevented by green tea catechins in a study in Italy (Bettuzzi et al. 2006). In addition to prevention of colorectal adenoma by green tea catechins, it is worthwhile to study whether green tea catechins inhibit overexpression of SET and CIP2A along with PP2A activity in cancer cells, as suitable targets for cancer prevention.

### Pancreatic cancer

Pancreatic cancer is a deadly disease that is usually diagnosed in the advanced stage and pancreatic cancer progression is negatively regulated by PP2A. Knockdown of SET or CIP2A increased PP2A activity and c-Myc degradation, and decreased the tumorigenic potential of pancreatic cancer cell lines both *in vitr*o and in vivo. Treatment with a SET inhibitor, OP449, significantly reduces xenografted tumor cell proliferation. Therefore, OP449 warrants further pre-clinical investigation as a potential pancreatic cancer therapeutic (Farrell et al. 2014). Splicing factor 3b subunit 1 (*SF3B1*) K700E mutation favored in vitro cell proliferation and in vivo tumor growth in pancreatic cancer cells. Further mechanistic studies identified that the *SF3B1* K700E mutation resulted in aberrant splicing of phosphoprotein phosphatase (PPP) 2R5A and led to an increase in c-Myc expression, which ultimately promoted the Warburg effect and tumor growth in pancreatic ductal adenocarcinoma (Yang et al. 2021).

## Discussion

There was a long-standing debate as to whether tumor promotion in mouse skin is mechanistically different from cancer progression in humans, because tumor promotion by TPA mainly produces benign tumors in mouse skin. It is now well accepted that tumor promotion in rodents by the okadaic acid class of compounds is linked to cancer progression in humans by the overexpression of PP2A inhibitors, via the same mechanism involving inhibition of PP2A activity (Fujiki et al. 2018a). Although the interactions of SET and CIP2A with various cellular factors is an attractive subject, we present some results only briefly. For example, SET forms a complex with the tumor suppressors, NM23-H1 and pp32 in the endoplasmic reticulum (Fan et al. 2003). Phosphorylation of Tau is regulated by PP2A, which in turn is modulated by SET. In Alzheimer’s disease, SET in the brain is translocated from the neuronal nucleus to the cytoplasm, where it inhibits PP2A activity and promotes abnormal phosphorylation of Tau (Arif et al. 2014). SET interacts with the Krüppel-associated box (KRAB)-associated co-repressor KAP1, and its overexpression results in sustained retention of KAP1 and Heterochromatin protein 1 on chromatin (Kalousi et al. 2015). The binding complex of SET and CIP2A with PP2A remains to be further investigated in cancer research. The inhibitory activity of PP2A by overexpression of SET and CIP2A increases hyper-phosphorylated proteins, including pS62-MYC and pAkt, and induces proinflammatory cytokines TNF-α and IL-1, which are presented as essential molecules in tumor promotion. How overexpression of SET and CIP2A proteins is induced in cancer cells remains to be investigated. However, based on evidence that activated fibroblasts obtained from old mice secrete inflammatory cytokines and TNF (Mahmoudi et al. 2019), it is necessary to consider the induction of proinflammatory cytokines caused by inhibition of PP2A activity due to chronic inflammation that is characteristic of aging (“inflammaging”) (Desdin-Micó et al. 2020) in the cancer microenvironment. Moreover, pyroptotic and necroptotic cells rupture and release many proinflammatory molecules associated with inflammation, including IL-1α, IL-33, and high mobility group box 1 (Newton et al. 2021). Although there is no direct evidence that SET and CIP2A are induced during inflammaging, overexpression of SET or CIP2A proteins in primary pre-cancerous tissues should be investigated in relation to the aging process. This will lead to cancer prevention and control within the framework of precision medicine.

## Conclusion

Inhibition of PP2A activity is a common mechanism of human cancer progression, so activation of PP2A activity leads to effective anticancer therapy.

## Data Availability

For original data, see references which we cited.
